# Intra-articular use of a medical device composed of hyaluronic acid and chondroitin sulfate (Structovial CS): effects on clinical, ultrasonographic and biological parameters

**DOI:** 10.1186/1756-0500-5-407

**Published:** 2012-08-04

**Authors:** Yves Henrotin, Jean-Philippe Hauzeur, Pierre Bruel, Thierry Appelboom

**Affiliations:** 1 Physical Therapy and Rehabilitation Department, Princess Paola Hospital, Vivalia, Marche-en-Famenne, Bone and Cartilage Research Unit, University of Liège Institute of Pathology, level 5, CHU Sart-Tilman, 4000, Liège, Belgium; 2 Department of Rheumatology, Erasme Hospital, University of Brussels, 808 Route de Lennik, 1070, Brussels, Belgium; 3 Laboratoires Pierre Fabre, 29 Avenue du Sidobre, 81 106, Castres Cedex, France

**Keywords:** Chondroitin sulphate, Hyaluronic acid, Knee osteoarthritis, Clinical trial, Osteoarthritis treatment, Ultrasonography, Biomarkers

## Abstract

**Background:**

This pilot open noncontrolled study was designed to assess the efficacy of intra-articular injections of a solution combining hyaluronic acid (HA) and chondroitin sulphate (CS) in the treatment of outpatients affected by knee osteoarthrosis.

**Findings:**

Thirty patients with knee OA have been included. The primary objective was to assess clinical efficacy as measured by pain and Lequesne’s index. Secondary objectives were to assess potential effect of the treatment on ultrasound parameters, safety and biomarkers of cartilage metabolism and joint inflammation. After a selection visit (V1), the study treatment was administered 3 times on a weekly basis (V2, V3, V4). Follow-up was planned 6 (V5) and 12 weeks (V6) after the first intra-articular injection. Efficacy results showed a reduction in mean pain at V3 and V6 and in functional impairment, the most marked changes being measured at the two follow-up visits (V5 and V6). Although statistical significance was not achieved due to small sample size, a clear tendency towards improvement was detectable for ultrasound assessments as well as biomarkers. Except for a mild injection site hematoma for which the drug causal relationship could not be excluded, no adverse effect of clinical relevance was recorded during the study.

**Conclusion:**

Although this pilot study was performed according to an open design only, the ultrasound as well as biomarkers changes strongly suggest a non-placebo effect. These preliminary results call now for a randomized controlled study to confirm the clinical relevance of the observed results.

**Trial registration:**

#ISRCTN91883031

## Findings

In the treatment of osteoarthritis (OA), it is now agreed that surgical procedures should be at least delayed, and even avoid inasmuch as possible.

Hyaluronic acid (HA) is a component of the synovial fluid, the lubricating effect of which is related to its viscoelastic properties. There is a large agreement that early manifestations of OA are related to changes in the viscoelasticity of the synovial fluid which account for a decrease in the protective action of the cartilage: such deterioration appears mainly due to a decrease in the concentration and molecular weight of synovial HA.

HA injections into the joint may compensate for this deficit in elasticity, thereby improving articular lubrication. There is a large body of data regarding HS biocompatibility, its toxicology as well as its metabolism [1-4].

Regarding HA clinical efficacy in knee OA, a number of studies are available, some of them performed according a double-blind placebo-controlled design [5-7]. According to the European League Against Rheumatism (EULAR) recommendations published in 2003, « there is evidence to support the efficacy of HA in the management of knee OA both for pain reduction and functional improvement » which may induce pain relief « for several months » [8].

Structovial CS (Pierre Fabre Médicament) is a medical device combining a chondroitin sulphate (CS) (30 mg/mL) and HA (12 mg/mL) to treat knee OA. The biocompatibility of both products has been assessed during Structovial CS development. The role of CS is twofold: i) optimizing HA’s rheological behaviour, due to specific interactions [9,10]; ii) regulating cartilage metabolism, as a substrate for polysulphated glycosaminoglycans synthesis as well as an inhibitor of catabolic cytokines and metalloproteinases synthesis (11-13).

The primary objective of this study was to provide some clinical, sonographic, biologic parameters of 3 weekly intra-articular injections of HA/CS in knees affected by OA over a period of 12 weeks.

Secondary objectives were to: i) assess the treatment effect on ultrasound (US) parameters; ii) analyze biomarkers known as related to cartilage metabolism and to joint inflammation; and iii) assess the treatment safety.

## Methods

This was a single-centre, open-label, uncontrolled study (Trial registration #ISRCTN91883031) designed to assess intra-articular injections of HA/CS in knee OA.

### Patients

Inclusion criteria were: male or female patients aged ≥ 45 and ≤ 80 years; suffering from internal and/or external femoro-tibial OA: meeting the criteria of the American College of Rheumatology (ACR) (14) (pain of the knee and crepitus on active motion or morning stiffness < 30 minutes or age >50 years); lasting for at least 6 months; pain ≥ 40 mm as measured on a visual analogue scale (VAS); stage II or III within the previous year according to the radiological classification of Kellgren and Lawrence [11]); OA deemed to justify a treatment with intra-articular HA according to the investigator; patient’s written, informed consent.

Non-inclusion criteria were related to any circumstances likely to interfere with the study treatment, namely: symptomatic femoro-patellar arthrosis or hip arthrosis on the same side, concomitant skeletal disease (Paget disease, rheumatoid arthritis, ankylosing spondylitis…); former or concomitant treatment (intra-articular corticosteroids, topical or oral NSAIDs, anti-arthritis slow acting treatment, recent surgery…); individual characteristics incompatible with a drug trial (pregnancy or lack of contraception, serious concomitant disease, participation in a clinical trial within the preceding 30 days…). Participation in the study could be prematurely withdrawn at the patient’s or investigator’s initiative, e.g. in case of a significant adverse event.

Patients were not allowed to take any pain relief medication (eg, NSAIDs, analgesics) or any OA therapy (eg, diacerein, glucosamine, CS). In the event of severe pain, and if necessary, patients were permitted to take 1 gram tablets of acetaminophen, 1 at a time, up to 4 times per day, with a minimum of 4 hours between tablets. If the recommended dosage of acetaminophen was insufficient, it was permitted to take a NSAID.

### Study schedule

The selection period ran from Day -21 to Day -1 (V1). The patients participated in the study from Day 0 to Day 84. The investigational drug was a sterile solution of HA/CS for intra-articular injections: each 2 mL injection contained 24 mg of HA and 60 mg of CS. It was injected on a weekly basis, on Days 0 (V2), 7 (V3, one week), and 14 (V4, 2 weeks). Then, Days 42 (V5, 6 weeks) and 84 (V6, 12 weeks) were for follow-up and end-of-study assessments, which brought to 6 the total of scheduled visits throughout the study.

### Study parameters

#### Clinical parameters

The main recorded parameters were a Visual Analog Scale (VAS) to measure spontaneous pain (from 0 = no pain to 100 = maximum pain), Lequesne’s Algo-Functional Knee Index [12], concomitant medication as well as adverse events if any; on V1 (first injection) and V6 (end-of-study follow-up). Overall assessment of improvement was assessed by the patient and by the investigator, using a VAS (from 0 = worsening to 100 = improvement). The clinical response was assessed at V5 and V6 using the criteria defined by the Osteoarthritis Research Society International (OARSI) [13]. On V6, the patients were asked about their satisfaction regarding the treatment.

#### Ultrasound parameters

An US examination of the target knee was performed with a Logic 9 (GE) device using a 10-15 mHz high resolution transducer. Joint fluid was assessed by a longitudinal scan of the suprapatellar recess: grade 0 = no, grade 1 = fluid only detected when an isometric quadricipital contraction is done by the patient, 2 = fluid even at rest [14]. Synovial thickness was measured on a longitudinal image of the suprapatellar recess with an extended knee, with a knee flexed at 545° and on a transversal scan of the lateral recess. The used value was the addition of the 3 measurements. A detection (and a quantification when positive) of any popliteal cyst was done: 0 = no, 1 = yes (in cc).

#### Biomarkers dosage

Several biomarkers have been directly measured in the serum using immunoassays and following the manufacturer instruction: inflammation markers [IL-6] (Biosource, Fleurus, Belgium), degradation [Coll2-1] (Artialis SA, Liège, Belgium) and synthesis [CPII] (IBEX technologies, Montreal, QC, Canada) of type II collagen, degradation of aggrecan [CS846] (IBEX technologies, Montreal, QC, Canada) and markers of oxidative stress [Coll2-1NO2] (Artialis SA, Liège, Belgium) were performed.

### Statistical and ethical considerations

As there was no control group, the efficacy analysis was mainly descriptive and there was no primary efficacy parameter. All tests performed were exploratory. All analyses were made using the statistical analysis software (SAS®) Version 9.1.3 on the UNIX operating system software. AEs were coded using the MedDRA 10.1. Quantitative parameters were described using the following descriptive statistics: number of patients, arithmetic mean, standard deviation (SD), minimum, median and maximum values, and first and third quartile.

Qualitative parameters were described using frequencies and percentages.

Efficacy parameters (absolute change from baseline) were analyzed by linear regression on baseline values. As there was only one treatment group, all analyses were exploratory.

For the statistical analysis, the date of first dose of study drug was considered relative Day 0 and the day before the first dose of study drug was considered Day -1. Relative days for assessments before, on, or after the first dose of study drug were calculated as follows): Relative Day = Date of Assessment – Date of First Dose (Day 0).

A sample size of 30 patients was considered sufficient as the study is explanatory.

The study protocol was approved on January 18 2008 by the Ethic Committee of Erasme Hospital, University of Brussels. The study was conducted in compliance with the Declaration of Helsinki and its amendments, the Good Clinical Practices (GCP 1996), and the ISO 14155 regulation.

## Results

### Disposition and description of patients

From March 10, 2008 to October 13, 2008 a total of 31 patients were screened/selected at the Hôpital Erasme in Brussels, Belgium. Of these, 30 patients were included in the study and were treated with HA/CS: all of them were included in the safety and efficacy analysis sets.

One patient, having completed Visit 5 (6 weeks), withdrew from the study on Day 101 for its personal convenience. No major protocol violation was reported within this study. The sex ratio of the included patients was 8 M/22 F, with a mean age [±SD] of 61.5 ± 9.4 years. Demographic data and baseline characteristics of included patients are summarized in Table 1. Regarding the patients joint condition, the median [range] time they had knee OA was 28 [5 - 195] months; most patients (20 [66.7%]) were assessed as Kellgren-Lawrence Grade II on the basis of their most recent X-ray. Knee OA history is summarized in Table 2. No other medical history or concomitant disease was identified as significant enough to interfere with the study assessments.

**Table 1:** Demographic and baseline characteristics of included patients

**Characteristic**	**Full Analysis Set (n = 30)**
Sex	n (%)
Female	22 (73.3)
Male	8 (26.7)
Age (years)	n (%)
mean ± SD	61.5 ± 9.4
<45 years	2 (6.7)
45 to 55 years	7 (23.3)
>55 to 65 years	8 (26.7)
>65 to 80 years	13 (43.3)
Weight (kg)	
mean ± SD	79.6 ± 12.9
Height (cm)	
mean ± SD	166.4 ± 9.9
Body mass index (kg/m²)	n (%)
mean ± SD	28.8 ± 4.0
<25 kg/m²	6 (20.0)
25 to 30 kg/m²	14 (46.7)
>30 kg/m²	10 (33.3)

**Table 2:** Osteoarthritis history in included patients

**Knee OA History**	**Full Analysis Set (n = 30)**
Target knee more painful at Visit 1	n (%)
Left	13 (43.3)
Right	17 (56.7)
Duration of knee OA, months	
mean ± SD	44.6 ± 48.7
median, range	28.0, 5-195
Family history	n (%)
Yes	7 (23.3)
Kellgren-Lawrence Grade in last X-ray	n (%)
Grade II	20 (66.7)
Grade III	10 (33.3)

### Efficacy parameters

Pain intensity decreased during the study: as compared to baseline, the change (mean ± SD) was -23.3 ± 22.51 at Visit 3 (one week) and -36.1 ±28.54 at Visit 6 (12 weeks). Linear regressions of the absolute changes were performed on the baseline values: the most significant changes from baseline were measured at Visit 5 (6 weeks) (p = 0.0008) and at Visit 6 (12 weeks) (p = 0.0042). The evolution of pain throughout the study is summarized in Table 3.

**Table 3:** Evolution of pain during the study (100-mm VAS: 0 = no pain, 100 = maximum pain)

	**Visit 3 (one week)(n = 30)**	**Visit 4 (2 weeks)(n = 30)**	**Visit 5 (6 weeks)(n = 29)**	**Visit 6 (12 weeks)(n = 30)**
Baseline, mm, mean ± SD	71.3 ± 14.71	71.3 ± 14.71	70.8 ± 14.73	71.3 ± 14.71
Post-baseline, mm, mean ± SD	48.0 ± 20.76	37.3 ± 21.87	31.3 ± 23.76	35.2 ± 24.59
Change, mm, mean ± SD	-23.3 ± 22.51	-34.0 ± 26.98	-39.5 ± 29.23	-36.1 ± 28.54

mm = millimetres; n = number of patients; SD = standard deviation.

Likewise, functional impairment as assessed by Lequesne’s index decreased during the study: as compared to baseline, the change (mean ± SD) was -1.34 ± 3.472 at Visit 3 and -3.40 ± 4.193 at Visit 6 (12 weeks). Linear regressions of the absolute changes were performed on the baseline values: the most significant changes from baseline were measured at Visit 5 (6 weeks) (p = 0.0031) and at Visit 6 (12 weeks) (p = 0.0012). The evolution of Lequesne’s algo-functional knee index is summarized in Table 4.

**Table 4:** Evolution of Lequesne’s algo-functional knee index during the study

	**Visit 3 (one week) (n = 29)**	**Visit 4 (2 weeks) (n = 29)**	**Visit 5 (6 weeks) (n = 28)**	**Visit 6 (12 weeks) (n = 29)**
Baseline, mm, mean ± SD	11.88 ± 2.966	11.88 ± 2.966	11.70 ± 2.849	11.88 ± 2.966
Post-baseline, mm, mean ± SD	10.53 ± 3.287	8.88 ± 2.770	8.21 ± 3.050	8.48 ± 3.483
Change mm, mean ± SD	-1.34 ± 3.472	-3.00 ± 3.036	-3.48 ± 3.420	-3.40 ± 4.193

mm = millimetres; n = number of patients; SD = standard deviation.

The patient and investigator assessment of global improvement changed only marginally throughout the study. The biggest difference in the VAS scores, for both the patients and investigators, was measured one week after the first study injection, but these differences were not significant.

A clinical response, as assessed by the OARSI criteria [13], was found in 23 patients (79%) at Visit 5 (6 weeks) and in 22 (73%) at Visit (12 weeks) 6.

On Visit 6 (12 weeks), most patients reported being “very satisfied” (n = 13 ; 45%) or “satisfied” (n = 7; 24%) with their treatment; only 4 patients (14%) patients claimed to be discontent.

The majority of patients exhibited a clinical response to treatment at Visit 5 (6 weeks) (79.3%) and Visit 6 (12 weeks) (73.3%).

Regarding ultrasound parameters, the results are summarized in Table 5. A reduction of the synovial thickness was found from Visit 2 (baseline) to Visit 6 (12 weeks), especially in patients displaying articular liquid at baseline; however, statistical significance was not achieved, probably because of small sample size. Likewise, fewer patients showed articular effusion at Visit 6 (12 weeks) (n = 13) as compared to Visit 2 (baseline) (n = 18), but the difference was not statistically significant.

**Table 5:** Ultrasound Parameters: Full Analysis Set

**Ultrasound Parameter**		**Visit 2 (baseline)**		**Visit 6(12 weeks)**
Articular effusion, n (%)		30		30
No liquids		12 (40.0)		17 (56.7)
Present only at isometric contraction		14 (46.7)		11 (36.7)
Present at rest and at isometric contraction		4 (13.3)		2 (6.7)
Height by isometric contraction, mm, mean ± SD	18	3.20 ± 1.682	13	3.34 ± 1.251
Synovial thickness in extension, mm, mean ± SD	30	1.72 ± 1.015	30	1.60 ± 0.972
Synovial thickness in 45° flexion, mm, mean ± SD	30	2.06 ± 1.196	30	1.83 ± 1.086
Synovial thickness in external recess, mm, mean ± SD	30	1.81 ± 0.993	30	1.57 ± 0.665
Synovial thickness total of 3 measurements, mm, mean ± SD	30	5.58 ± 2.591	30	5.00 ± 2.139
Popliteal cyst present, n (%)	29	6 (20.7)	30	7 (23.3)
Popliteal cyst volume, mm^3^, mean ± SD	4	471.09 ± 869.230	6	2924.73 ± 3783.539

mm = millimeter; n = number of patients; SD = standard deviation; Note: Percentages are based on available information.

The results obtained for biomarkers are summarized in Table 6. Mean values of Coll2-1, Coll2-1NO2 and CPII decreased between Visit 2 (baseline) and Visit 6 (end of the study, 12 weeks). To measure the linear dependency between biomarkers and pain intensity, correlation coefficients were researched between the absolute change of each biomarker and the absolute change of pain from Visit 2 (baseline) and V6 (12 weeks). The coefficients of correlation were mostly negative indicating that more the biomarker level change is high, less the pain change is important. Of note, the results observed on IL-6, with a dramatic reduction from 5825 ± 21720 pg/mL (baseline) to 162 ± 405 pg/mL.

**Table 6:** Biomarkers: Full Analysis Set

**Biomarker**	**Coll 2-1 (nM) n = 21**	**Coll 2-1 NO2 (nM) n = 21**	**CS-846 (ng/mL) n = 20**	**CP II (ng/mL) n = 21**	**IL-6 (pg/mL) n = 18**
		**mean ± SD**			
V2 (baseline)	127 ± 62	0.44 ± 0.29	92 ± 24	1040 ± 518	5825 ± 21720
V6 (D84)	116 ± 37	0.38 ± 0.18	93 ± 21	1000 ± 646	162 ± 405
Change	-11 ± 78	-0.06 ± 0.41	1 ± 17	-41 ± 865	-5663 ± 21769
	**correlation coefficients (biomarkers change vs pain change)**				
Pearson	-0.395	-0.412	-0.021	-0.469	-0.086
	p = 0.0766	p = 0.0637	p = 0.9296	p = 0.0319	p = 0.7354
Spearman	-0.274	-0.262	0.026	-0.319	0.151
	p = 0.2287	p = 0.2512	p = 0.9122	p = 0.1584	p = 0.5500

mL = milliliter; n = number of patients; ng = nanogram; nM = nanomolar; pg = picogram; SD = standard deviation.

Note: Only patients with both a baseline value and a time point value are summarized at Visit 6; Correlation coefficients between the absolute change of each biomarker and the absolute change of pain from V2 and V6.

### Safety parameters

No severe adverse event was reported throughout the study.

Of the 30 patients included in the safety analysis, 4 reported an adverse event: injection site haematoma (n = 1, 3.3%), wrist fracture (n = 1, 3.3%), arthralgia (n = 1, 3.3%), and venous stasis (n = 1, 3.3%). Of mild intensity, the haematoma was the only reported adverse event for which a drug causal relationship was not excluded by the investigator.

No abnormality of clinical relevance was reported in vital or physical signs monitored during the study.

## Discussion

The purpose of this open study was to assess Structovial CS (Pierre Fabre Médicament), a solution combining chondroitin sulphate (CS) (30 mg/mL) and HA (12 mg/mL) and administered by intra-articular injections, in 45 to 80-year old patients suffering from femoro-tibial OA.

All enrolled patients received 3 intra-articular injections of a solution of HA/CS over a 3-week period, and were assessed in 6 clinic visits, up to 10 weeks after their last injection. Efficacy was assessed through measure of pain, functional impairment, clinical response, ultrasound and biomarkers.

Both pain intensity and functional impairment decreased during the study. The most significant changes for both parameters were observed at 6 and 12 weeks after the first study injection.

The patient and investigator assessment of global improvement changed only marginally throughout the study. The biggest difference in the VAS scores, for both the patients and investigators, was measured one week after the first study injection, but statistical significance was not achieved.

The majority of patients exhibited a clinical response to treatment at 6 weeks (79.3%) and 12 weeks after the first study injection (73.3%).

No statistically significant changes in ultrasound parameters were seen throughout the study, although an improvement was found in term of a reduction in number of effusion and in term of synovial thickness. With a larger sample size this probable effect on the synovial inflammation could be demonstrated.

The 5 measured biomarkers displayed a high variability although they tended to decrease in a consistent way throughout the study.

No serious adverse events, no adverse event leading to study discontinuation, and no deaths were reported during the study.

A total of 4 (13.3%) adverse events (AEs) were reported throughout the study: injection site haematoma, wrist fracture, arthralgia, and venous stasis. The injection site haematoma was of mild intensity. For this AE, the investigator did not exclude a relation to the study drug.

No other change of clinical relevance was observed in physical examination or vital signs.

On the basis of these results, the discussion should be balanced. In this non controlled study, the improvement in clinical parameters (pain intensity, functional impairment) was not clearly greater than that which could be induced by a placebo in a controlled study. On the other hand, the structural as well as biomarkers changes suggest a non-placebo effect, as lack of statistical significant for both “objective” parameters is most probably a consequence of small sample size. In particular, biomarkers changes appeared quite consistent although statistically not significant, with a decrease in Coll2-1 (degradation marker) and in IL-6 and Coll2-1NO2 (markers of oxidative stress and of inflammation). In a previous study, Hosigawa et al. already showed that intra-articular injection of hyaluronan was associated with a reduction in biomarkers in synovial fluid, suggesting that HA could help maintain normal cartilage metabolism at least in patients at an early stage of OA and with limited synovitis [15]. The biomarkers are the reflect of cartilage degradation, then directly correlated with the disease activity (i.e. inflammation and pain). The change in biomarker levels due to the medical device over time can be linked to the change in disease activity. By the way, the more important is the change in biomarkers, the more important is the effect on pain.

Overall, the results of this pilot study are consistent with a favourable benefit/risk ratio of the medical device used, but they strongly call for undergoing now a randomized clinical trial with the required statistical power.

## Conclusions

Intra-articular injections of HA is a well-established therapy for the treatment of knee OA. The study was designed to support the clinical efficacy and safety data on the use of HA when combined with CS for intra-articular injections.

The injection pattern of 1 injection weekly over 3 weeks is the current treatment pattern of most clinical studies of HA to date and is the treatment pattern of HA solutions available on the market. The present study followed this treatment regimen.

Although limited by a lack of control group as well as small sample size, the results of this pilot study suggest that intra-articular treatment with HA/CS (Structovial CS, Pierre Fabre Médicament) is effective and safe in patients with knee OA. These results should be confirmed now by a randomised clinical trial on a bigger sample size.

## Abbreviations

AE: adverse event; CS: chondroitin sulfate; HA: hyaluronic acid; IL: interleukine; OA: osteoarthritis; US: ultrasound; VAS: visual analog scale.

## Competing interests

PB is an employee of Pierre Fabre pharmaceutical company. YH is the founder of spin-off of the University of Liège, Artialis sa. TA has no direct conflict of interest; JPH has no direct conflict of interest.

## Authors’ contributions

YH: paper writing, data interpretation, biomarker analysis. TA: study coordination, patients inclusion, data interpretation, JPH: patient inclusion, ultrasound analysis, data interpretation; PB: paper writing, data interpretation. All authors read and approved the final manuscript.

An open-label study in patients with gonarthrosis.

## References

[B1] Conte A de Bernardi M Palmieri L Lualdi P Mautone G Ronca G Metabolic fate of exogenous chondroitin sulfate in man Arzneimittelforschung 1991 41 768 772 1772467

[B2] Conte A Palmieri L Segnini D Ronca G Metabolic fate of partially depolymerized chondroitin sulfate administered to the rat Drugs Exp Clin Res 1991 17 27 33 1914833

[B3] Conte A Volpi N Palmieri L Bahous I Ronca G Biochemical and pharmacokinetic aspects of oral treatment with chondroitin sulfate Arzneimittelforschung 1995 45 918 925 7575762

[B4] Palmieri L Conte A Giovannini L Lualdi P Ronca G Metabolic fate of exogenous chondroitin sulfate in the experimental animal Arzneimittelforschung 1990 40 319 323 2112001

[B5] Huskisson EC Donnelly S Hyaluronic acid in the treatment of osteoarthritis of the knee Rheumatology (Oxford) 1999 38 602 607 10.1093/rheumatology/38.7.602 10461471

[B6] Lohmander LS Dalen N Englund G Hamalainen M Jensen EM Karlsson K Odensten M Ryd L Sernbo I Suomalainen O Tegnander A Intra-articular hyaluronan injections in the treatment of osteoarthritis of the knee: a randomised, double blind, placebo controlled multicentre trial. Hyaluronan Multicentre Trial Group Ann Rheum Dis 1996 55 424 431 10.1136/ard.55.7.424 8774159 PMC1010204

[B7] Puhl W Bernau A Greiling H Kopcke W Pforringer W Steck KJ Zacher J Scharf HP Intra-articular sodium hyaluronate in osteoarthritis of the knee: a multicenter, double-blind study Osteoarthr Cartil 1993 1 233 241 10.1016/S1063-4584(05)80329-2 15449510

[B8] Jordan KM Arden NK Doherty M Bannwarth B Bijlsma JW Dieppe P Gunther K Hauselmann H Herrero-Beaumont G Kaklamanis P Lohmander S Leeb B Lequesne M Mazieres B Martin-Mola E Pavelka K Pendleton A Punzi L Serni U Swoboda B Verbruggen G Zimmerman-Gorska I Dougados M EULAR Recommendations 2003: an evidence based approach to the management of knee osteoarthritis: Report of a Task Force of the Standing Committee for International Clinical Studies Including Therapeutic Trials (ESCISIT) Ann Rheum Dis 2003 62 1145 1155 10.1136/ard.2003.011742 14644851 PMC1754382

[B9] Kato Y Mukudai Y Okimura A Shimazu A Nakamura S Effects of hyaluronic acid on the release of cartilage matrix proteoglycan and fibronectin from the cell matrix layer of chondrocyte cultures: interactions between hyaluronic acid and chondroitin sulfate glycosaminoglycan J Rheumatol Suppl 1995 43 158 159 7752124

[B10] Nishimura M Yan W Mukudai Y Nakamura S Nakamasu K Kawata M Kawamoto T Noshiro M Hamada T Kato Y Role of chondroitin sulfate-hyaluronan interactions in the viscoelastic properties of extracellular matrices and fluids Biochim Biophys Acta 1998 1380 1 9 10.1016/S0304-4165(97)00119-0 9545510

[B11] Kellgren J Lawrence D Radiological assessment of osteoarthritis Ann Rheum Dis 1957 16 494 502 10.1136/ard.16.4.494 13498604 PMC1006995

[B12] Lequesne MG Mery C Samson M Gerard P Indexes of severity for osteoarthritis of the hip and knee. Validation--value in comparison with other assessment tests Scand J Rheumatol Suppl 1987 65 85 89 3479839 10.3109/03009748709102182

[B13] Pham T Van Der Heijde D Lassere M Altman RD Anderson JJ Bellamy N Hochberg M Simon L Strand V Woodworth T Dougados M Outcome variables for osteoarthritis clinical trials: The OMERACT-OARSI set of responder criteria J Rheumatol 2003 30 1648 1654 12858473

[B14] Hauzeur JP Mathy L De Maertelaer V Comparison between clinical evaluation and ultrasonography in detecting hydrarthrosis of the knee J Rheumatol 1999 26 2681 2683 10606382

[B15] Hasegawa M Nakoshi Y Tsujii M Sudo A Masuda H Yoshida T Uchida A Changes in biochemical markers and prediction of effectiveness of intra-articular hyaluronan in patients with knee osteoarthritis Osteoarthr Cartil 2008 16 526 529 10.1016/j.joca.2007.09.014 17951079

